# High LACE index scores are associated with disproportionate excess deaths in hospital amongst patients with COVID-19

**DOI:** 10.1007/s11739-022-03015-8

**Published:** 2022-06-22

**Authors:** David Fluck, Christopher Henry Fry, Jonathan Robin, Thang Sieu Han

**Affiliations:** 1 Department of Cardiology, Ashford and St Peter’s Hospitals NHS Foundation Trust, Guildford Road, Chertsey, Surrey KT16 0PZ UK; 2 School of Physiology, Pharmacology and Neuroscience, University of Bristol, Bristol, BS8 1TD UK; 3 Department of Medicine, Ashford and St Peter’s Hospitals NHS Foundation Trust, Guildford Road, Chertsey, Surrey KT16 0PZ UK; 4 Department of Endocrinology, Ashford and St Peter’s Hospitals NHS Foundation Trust, Guildford Road, Chertsey, Surrey KT16 0PZ UK; 5 Institute of Cardiovascular Research, Royal Holloway, University of London, Egham, Surrey TW20 0EX UK

**Keywords:** Healthcare quality, Health promotion, Practice management

## Abstract

Risk factors for COVID-19-related outcomes have been variably reported. We used the standardised LACE index to examine admissions and in-hospital mortality associated with COVID-19. Data were collected in the pre-pandemic period (01-04-2019 to 29-02-2020) from 10,173 patients (47.7% men: mean age ± standard deviation = 68.3 years ± 20.0) and in the pandemic period (01-03-2019 to 31-03-2021) from 12,434 patients. With the latter, 10,982 were without COVID-19 (47.4% men: mean age = 68.3 years ± 19.6) and 1452 with COVID-19 (58.5% men: mean age = 67.0 years ± 18.4). Admissions and mortality were compared between pre-pandemic and pandemic patients, according to LACE index. Admission rates rose disproportionately with higher LACE indices amongst the COVID-19 group. Mortality rates amongst the pre-pandemic, pandemic non-COVID-19 and COVID-19 groups with LACE index scores < 4 were 0.7%, 0.5%, 0%; for scores 4–9 were 5.0%, 3.7%, 8.9%; and for scores ≥ 10 were: 24.2%, 20.4%, 43.4%, respectively. The area under the curve receiver operating characteristic for predicting mortality by LACE index was 76% for COVID-19 and 77% for all non-COVID-19 patients. The risk of age and sex-adjusted mortality did not differ from the pre-pandemic group for COVID-19 patients with LACE index scores < 4. However, risk increased drastically for scores from 4 to 9: odds ratio = 3.74 (95% confidence interval = 2.63–5.32), and for scores ≥ 10: odds ratio = 4.02 (95% confidence interval = 3.38–4.77). In conclusion, patients with LACE index scores ≥ 4 have disproportionally greater risk of COVID-19 hospital admissions and deaths, in support of previous studies in patients without COVID-19. However, of importance, our data also emphasise their increased risk in patients with COVID-19. Because the LACE index has a good predictive power of mortality, it should be considered for routine use to identify high-risk COVID-19 patients.

**Supplementary Information:**

The online version contains supplementary material available at 10.1007/s11739-022-03015-8.

## Introduction

Coronavirus disease (COVID-19) has contributed to a high proportion of deaths in adults world-wide from the beginning of the pandemic in 2019 [1]. Since then, complications of COVID-19, including symptom severity, intensive care admission and mortality, have been associated with a wide range of risk factors. These include: male gender [2–4], obesity [4, 5], underlying chronic health conditions [3–5], learning difficulties [6], and older age [3, 4, 6, 7]. Also, included are deprivation [2, 4], unmarried status, as an immigrant from a low or middle-income country [2], people living in multi-occupancy dwellings [6] and ethnic minority groups [6, 8]. However, these individual risk factors have been variably reported between studies, so that currently there exists no consensus for the use of any standardised health index to predict the risk of complications and outcomes from COVID-19. Consequently, it is difficult, or impossible, to utilise existing data to compare clinical performances between studies.

The LACE index (Length of stay; Acuity of admission; Charlson co-morbidity index; Emergency department visits) scoring tool has been developed and validated to predict health outcomes, such as short-term mortality, readmission after a hospital discharge [9] and in-hospital mortality [10, 11]. This standardised index has been used widely in research and clinical practice for a number of years in different populations and conditions [10–15]. In this study, we hypothesised that patients with high LACE index scores might be at increased risk of death from COVID-19 infection. We have thus used the LACE index to examine in-hospital mortality during the COVID-19 pandemic, in both patients admitted with COVID-19 and those admitted with general medical conditions (non-COVID-19), and in comparison to a reference group, namely patients admitted with general medical conditions during the immediately preceding year.

## Materials and methods

### Study design, participants and setting

We analysed prospectively collected data of 22,644 consecutive unplanned admissions to a single NHS hospital (Ashford and St Peter’s NHS Foundation Trust, Surrey, UK). There were 37 cases (0.16%) for whom LACE index scores could not be calculated. This left 22,607 patients for analysis, comprising a group of 10,173 patients admitted before the COVID-19 pandemic (1 April 2019 to 29 February 2020), and 12,434 patients admitted during the pandemic (1 March 2020 to 31 March 2021). The pandemic group itself comprised 10,982 with general medical conditions (without COVID-19) and 1,452 with COVID-19 [16].

### Measurements

Clinical data were recorded including age, sex and comorbidities (coded according to the international classification of diseases, ICD-11) [17] for calculation of the Charlson co-morbidity index [18]. The LACE index was calculated from Length of stay (score range 0–7), Acuity of admission (score range 0 or 3), Charlson co-morbidity index (score range 0–5), Emergency department visits (score range 0 or 4). The LACE index scale therefore ranges from 0 to 19 [19].

### Categorisation of variables

The LACE index was categorised into two-point intervals, except for the lowest category (range 0–3) and highest category (range 14–19), for the initial exploration of the relationship between the LACE index and the distributions of admissions and mortality. This was followed by the creation of three LACE index categories based on the previous levels: scores < 4, 4–9, and ≥ 10 [10–12].

### Statistical analysis

Chi-square tests were used to examine differences between categorical variables including rates of admission and in-hospital mortality. Analysis of variance (ANOVA) was used to assess differences between continuous variables, including age, amongst patients admitted before and during the pandemic. Receiver operating characteristic (ROC) curves were constructed to determine the area under the curve (AUC) for the LACE index, as a predictor of in-hospital mortality. Logistic regression was used to assess the risk of in-hospital mortality (dependent variables) in patients admitted during the COVID-19 pandemic compared to those admitted before the pandemic (reference group), according to different LACE index categories. Odds ratios (OR) and 95% confidence intervals (CI) are presented as two models; model 1: unadjusted, and model 2: adjusted for age and sex. Analyses were performed using IBM SPSS Statistics, v25.0 (IBM Corp., Armonk, NY).

## Results

### General description

**Fig. 1:**
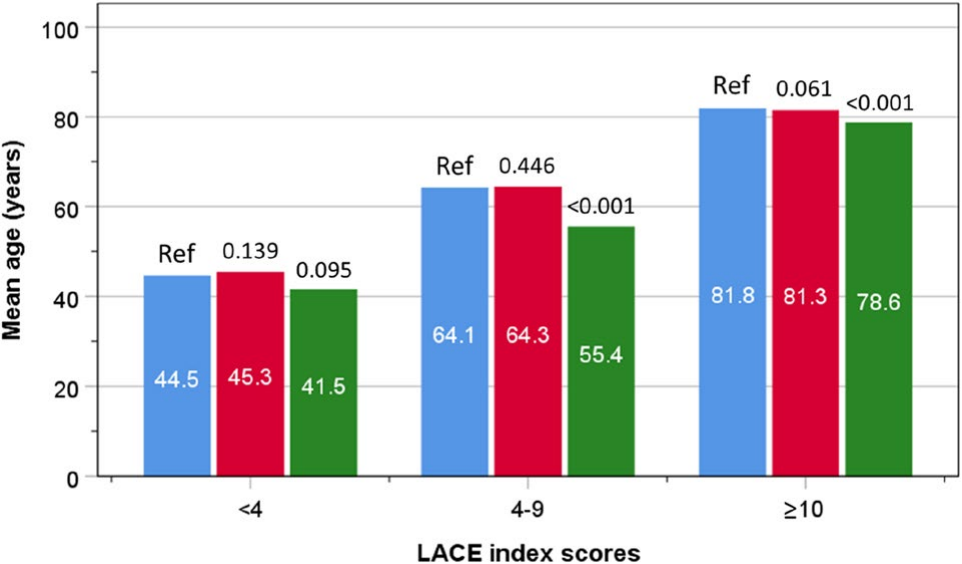
Mean age amongst patients admitted before the pandemic (blue bars) and during the pandemic with (green bars) or without COVID-19 (red bars), according to LACE index categories (color figure online)

**Fig. 2:**
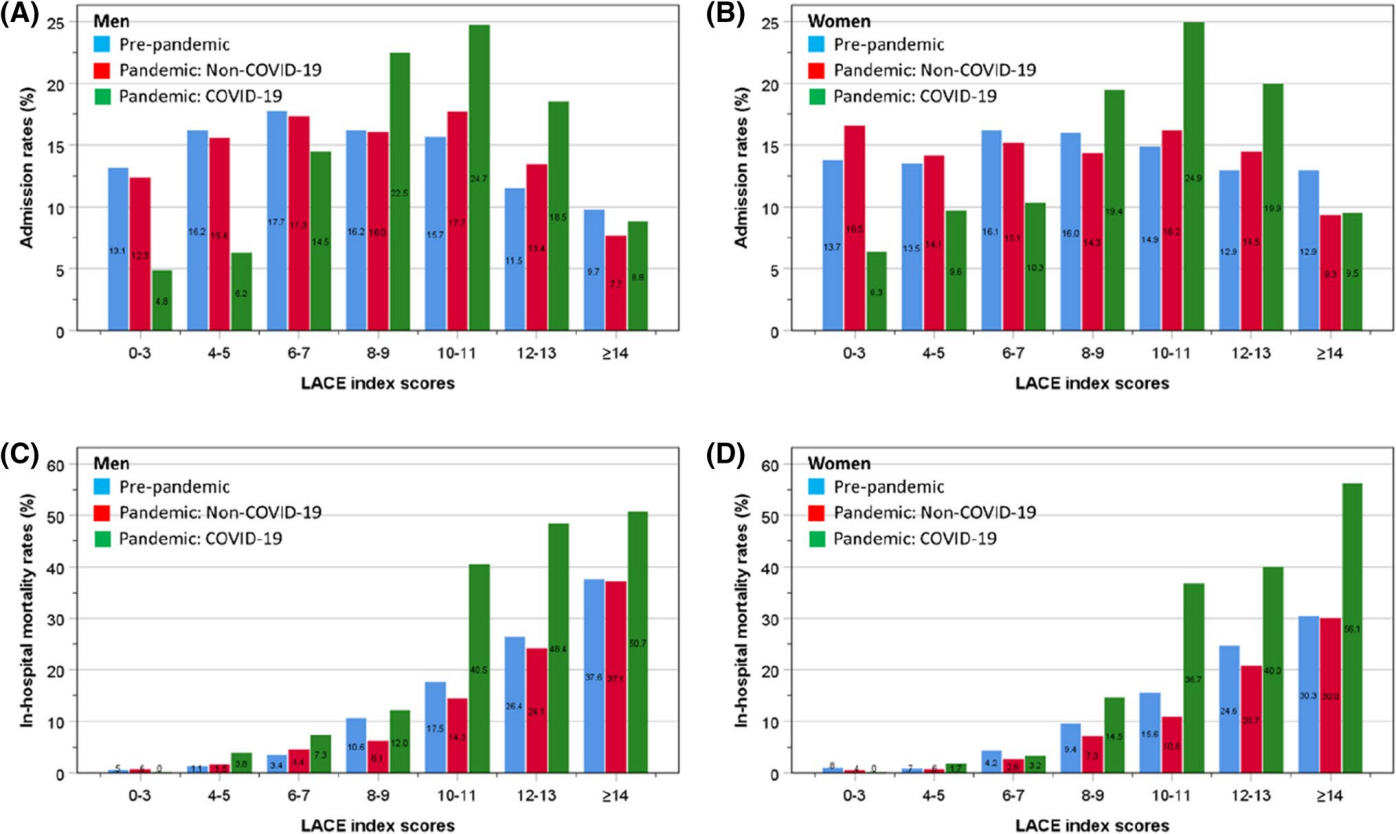
Admission rates in men (**A**) and women (**B**), and mortality rates in men (**C**) and women (**D**) before and during the pandemic according to LACE index categories

Data were analysed from a total of 22,607 patients (48.3% men) admitted either before the COVID-19 pandemic (1 April 2019 to 29 February 2020), or during the pandemic (1 March 2020 to 31 March 2021). There were 10,173 patients admitted in the pre-pandemic period with a mean (SD) age of 68.3 years (20.0), 10,982 non-COVID-19 patients in the pandemic period with a mean age of 68.3 years (19.6), and 1452 with COVID-19, with a mean age of 67.0 years (18.4). There were 13.4, 46.5, and 39.9% of patients within the LACE index categories of < 4, 4–9 and ≥ 10 respectively. Overall, the proportion of deaths in hospital was 7.9% (Supplementary Table 1). In addition, there were no age differences between groups of patients admitted before the pandemic and those admitted during the pandemic without COVID-19 (*P* = 0.868). However, patients admitted with COVID-19 were younger than both the pre-pandemic (*P* = 0.014) and pandemic non-COVID-19 (*P* = 0.017) groups. Compared with patients admitted before the pandemic, patients with COVID-19 had a similar age in the lowest LACE index category, but were younger amongst the higher categories (Fig. 1),

Based on the LACE index categories at two-point intervals (but including the two extreme categories of 0–3 and ≥ 14), men and women had similar admission rates between the pre-pandemic and pandemic non-COVID-19 patients for any given LACE index score (Fig. 2A, B). By contrast, in both sexes, there were relatively smaller proportions of patients admitted with COVID-19 who had lower range of LACE index score (< 8). However, COVID-19 admissions began to exceed those of non-COVID-19 patients for those who had a LACE index score ≥ 8. An exception was for the highest category (LACE index score ≥ 14) where the rates of admission for COVID-19 were similar to those of the non-COVID-19 groups. Overall, the patterns of admissions for each of the three study groups within each LACE index category were almost identical for men and women (Fig. 2A, B). Based on the three LACE index categories (scores < 4, 4–9 and ≥ 10), compared to the pre-pandemic reference group, the pandemic non-COVID-19 group had similar distributions of gender and LACE index categories, and lower in-hospital mortality rates (7.6 versus 6.0%) (Table 1). The proportion of male admissions was significantly (*χ*^2^ = 65.8, *P* < 0.001) higher amongst those with COVID-19 (58.5% men), compared both to those admitted without COVID-19 during the pandemic (47.4% men), and those during the pre-pandemic period (47.7% men). Amongst patients admitted with COVID-19, there was a higher proportion with a LACE index ≥ 10 (53.0 versus 38.8%), and in-hospital deaths (24.5 versus 7.6%) in comparison to the pre-pandemic and non-COVID-19 pandemic groups (Table 1).

**Table 1:** Distribution of characteristics, admissions and mortality according to period of study

	Pre-pandemic (*n* = 10,173)		Pandemic non-COVID-19 (*n* = 10,982)		Pandemic COVID-19 (*n* = 1452)	
	Mean	SD	Mean	SD	Mean	SD
Age (years)	68.3	20.0	68.3	19.6	67.0	10.4
Sex distribution	*n*	%	*n*	%	*n*	%
Men	4854	47.7	5229	47.5	850	58.5
Women	5319	52.3	5790	52.5	602	41.5
LACE index scores						
< 4	1368	13.4	1597	14.5	79	5.4
4–9	4855	47.7	5061	45.9	604	41.6
≥ 10	3950	38.8	4324	39.2	769	53.0
Mortality in hospital	772	7.6	662	6.0	356	24.5

### LACE index and mortality

**Fig. 3:**
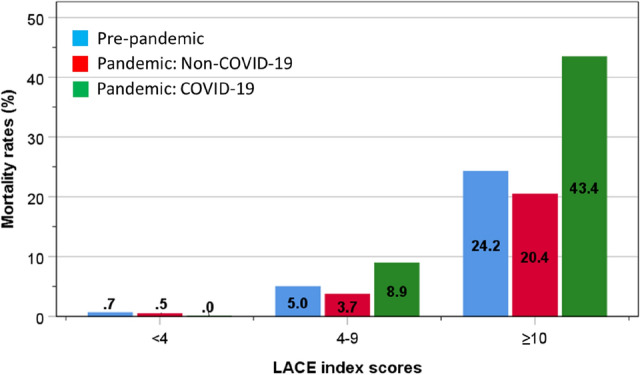
Mortality rates amongst patients admitted before the pandemic and during the pandemic with or without COVID-19, according to LACE index categories

Based on the two-point intervals of the LACE index scores, there were similarities for in-hospital mortality between groups, rising progressively with increasing LACE index scores. Mortality rates were also similar amongst patients admitted before and during the pandemic without COVID-19 for any given LACE index score. By contrast, mortality rates for COVID-19 patients with low LACE index score (< 8) were slightly higher than those in non-COVID patients, but amongst those with LACE index scores ≥ 8, the mortality rates rose to a much greater extent, peaking at over 50% amongst those with LACE index scores of ≥ 14 in both sexes. The patterns of mortality for each study group within each LACE index category were similar for men and women (Fig. 2C, D). In-hospital mortality rates were similarly low for pre-pandemic, pandemic non-COVID-19 and COVID-19 groups: 0.7 vs 0.5 vs 0% amongst lowest (< 4) LACE index scores. However, these rose disproportionately amongst COVID-19 patients: 5.0 vs 3.7 vs 8.9% amongst intermediate (4–9) LACE index scores; and 24.2 vs 20.4 vs 43.4% amongst LACE index scores ≥ 10 (Fig. 3).

After adjustment for age and sex, compared to the pre-pandemic (reference) period, in-hospital mortality did not differ for those admitted during the pandemic without COVID-19 for those with a LACE index score < 10. However, mortality was significantly lower for those with a score ≥ 10: adjusted OR = 0.76 (95% CI 0.67–0.86). The risk of in-hospital mortality also did not differ from the reference group for patients with COVID-19 who had a LACE index score < 4. However, in-hospital death in patients admitted with COVID-19 was significantly greater amongst those with a LACE index score between 4 and 9: adjusted OR = 3.74 (95% CI 2.63–5.32) and rose further amongst those with a LACE index score ≥ 10: adjusted OR = 4.02 (95%CI 3.38–4.77) (Table 2).

**Table 2:** Risk of death in with or without COVID-19 during the pandemic compared to patients before the pandemic according to LACE index scores

	Risk of in-hospital mortality with reference to pre-pandemic admissions					
	Pandemic: non-COVID-19 patients			Pandemic: COVID-19 patients		
	OR	95% CI	*P*	OR	95% CI	*P*
Unadjusted						
LACE index scores < 4	1.07	0.29–4.00	0.919	–^a^	–^a^	0.997
LACE index scores 4–9	0.80	0.64–1.00	0.054	2.35	1.68–3.29	< 0.001
LACE index scores ≥ 10	0.76	0.67–0.86	< 0.001	3.75	3.17–4.44	< 0.001
Age and sex-adjusted						
LACE index scores < 4	1.07	0.29–4.01	0.921	–^a^	–^a^	0.997
LACE index scores 4–9	0.80	0.63–1.01	0.057	3.74	2.63–5.32	< 0.001
LACE index scores ≥ 10	0.76	0.67–0.86	< 0.001	4.02	3.38–4.77	< 0.001

^a^Odd ratios could not be estimated due to low mortality rates amongst COVID-19 patients (see Supplementary Fig. 1)

### Ability of the LACE index to predict mortality

**Fig. 4:**
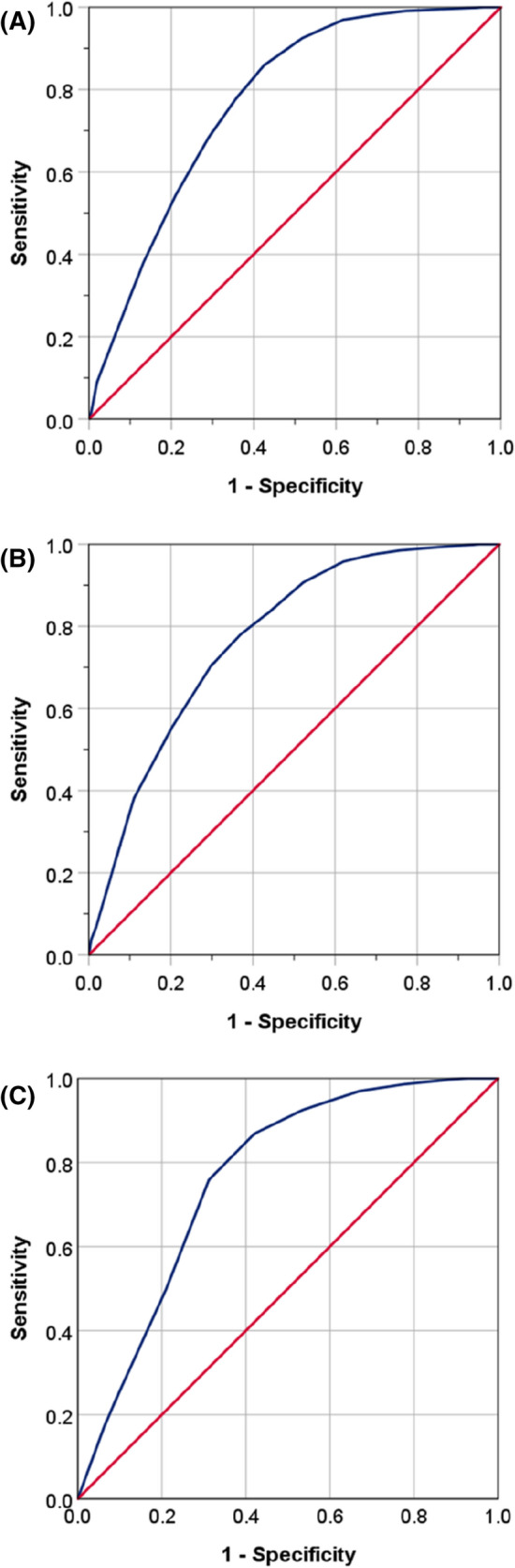
ROC curves analysis in the prediction of mortality from LACE index scores in patients admitted before the pandemic (**A**), and during the pandemic without (**B**) and with COVID-19 (**C**)

ROC analysis showed comparable performance of LACE index scores in the prediction of in-hospital mortality amongst all three study groups. The AUC was 77.3% (95% CI 75.9–78.7%, *P* < 0.001) for the pre-pandemic group (Fig. 4A); 77.2% (95% CI 75.7–78.8%, *P* < 0.001) for the pandemic non-COVID-19 group (Fig. 4B) and 76.4% (95% CI 73.9–79.0%, *P* < 0.001) for the COVID-19 group (Fig. 4C).

## Discussion

### Key findings

The LACE index has become a universally accepted index for predicting early readmissions and mortality in patients without COVID-19, for which the majority of findings were published before the pandemic. As far as we are aware, however, this is the first large study to use the LACE index in the study of admission and mortality rates for patients with COVID-19. A number of important findings emerged from this study: (i) a high LACE index score is a risk factor for increased hospital admissions with or without COVID-19, (ii) amongst patients with high LACE index scores, COVID-19 is associated with disproportionately higher rates of in-hospital mortality, and (iii) the LACE index has a similar predictive power of in-hospital mortality in patients admitted with COVID-19 to patients admitted with a general medical condition (non-COVID-19). The standardised LACE index scoring tool, routinely used in clinical settings, should therefore be considered for future routine use to identify high-risk patients with COVID-19 and enable inter-study comparison of clinical research and practice.

### Admission rates according to LACE index scores

The present study extended to COVID-19, showed evidence for a relationship between the LACE index and health outcomes. The observation that over half (53%) of patients admitted with COVID-19 had a LACE index score ≥ 10 suggest most of this group of patients had underlying poor health, although they were significantly younger than non-COVID patients. The rates of admission did not increase in the highest LACE index category (score ≥ 14) and suggest a survival bias, i.e. a proportion of those with poorest health or oldest age may have died before being admitted to hospital.

The LACE index has been shown extensively to relate to a number of outcomes including frequent early readmissions, and mortality whether in-hospital or after a discharge from hospital, in different study populations and admitted with a variety of non-COVID-19 medical conditions [10–15]. Unlike existing binary variables used to relate COVID-19 outcomes with variables, such as gender, ethnicity, obesity and learning difficulties, the LACE index has a wide range of scale (0–19 points). This allows more detailed analyses including the use of ROC curves to assess the power of prediction, and multiple thresholds to be examined in relation to COVID-19 outcomes. For example, the higher proportion of men admitted in the COVID-19 group is consistent with previous reports [2–4].

### Mortality according to LACE index scores

The proportionately excessive risk of death from COVID-19 amongst patients with high LACE index scores has not been reported in the existing literature. Furthermore, there was a stepwise increment in the risk of death with increasing LACE index scores. Remarkably, there were no deaths amongst the 79 patients admitted with COVID-19 and who had a low LACE index score of < 4. However, compared to patients without COVID-19, the risk of death from COVID-19 rose disproportionately with increasing LACE index scores. This emphasises that the excess mortality associated with COVID-19 is accentuated by underlying poor health.

In addition, we have also shown a lower risk of mortality amongst non-COVID-19 patients admitted during the pandemic compared to those admitted before the pandemic, particularly if in the highest LACE index category. These findings are in consistent with our previous report [20] and suggest that the standard of care-quality of patients admitted with general medical conditions continued to be maintained, or even increased, during the pandemic.

### Ability of the LACE index to predict mortality

The use of ROC curve analysis showed that the LACE index had a predictive power of in-hospital mortality amongst patients admitted with COVID-19 (AUC = 76%) similar to that amongst patients admitted with a general medical condition (AUC = 77%). These figures are also comparable to those reported previously (AUC = 66–83%) [10, 21–23]. This observation therefore supports the use of the LACE index scoring tool to identify high-risk patients admitted to hospital with COVID-19.

### Strengths and limitations

The strengths of this study lie in the large number of patients with very few missing cases, and the use of a reference group admitted immediately prior to the pandemic, and in the same centre, for comparison. Thus, the methods of data collection were consistent, including construction of LACE index scores. Thus, analyses could be conducted more readily with robust statistical techniques including ROC curves and logistic regression, with appropriate adjustments. This study focussed on the COVID-19 pandemic wave 1 and wave 2, which included the delta and alpha variants. The recent omicron variant may pose a different outcome. Similarly, caution should be taken with other variants in other parts of the world.

In conclusion, we have demonstrated that patients with high LACE index scores have disproportionally greater risk of COVID-19 admissions to hospital and related deaths. This index should therefore be considered for routine use in the identification of high-risk patients with COVID-19.

## Supplementary Information

Below is the link to the electronic supplementary material.

Supplementary file1 (DOCX 23 KB)

## Abbreviations

ANOVA: Analysis of variance
AUC: Area under the curve
CI: Confidence interval
COVID-19: Coronavirus disease
ICD: International classification of diseases
OR: Odds ratio
LACE: Length of stay; Acuity of admission; Charlson co-morbidity index; Emergency department visits
ROC: Receiver operating characteristic
