# Supplementary material for: High LACE index scores are associated with disproportionate excess deaths in hospital amongst patients with COVID-19
Source: Intern Emerg Med. 2022 Jun 22;17(7):1891–7. doi: 10.1007/s11739-022-03015-8 (PMC9216304; doi:10.1007/s11739-022-03015-8)
Supplement: Supplementary file 1 — Supplementary file1 (DOCX 23 KB) [file 11739_2022_3015_MOESM1_ESM.docx]

**Supplementary Table 1.** Characteristics of 22,607 patients admitted before the COVID-19 pandemic (1^st^ April 2019 to 29^th^ February 2020), and during the pandemic (1^st^ March 2020 to 31^st^ March 2021).

| **Sex** | n | % |
| --- | --- | --- |
| Men | 10911 | 48.3 |
| Women | 11696 | 51.7 |
| **LACE index** |  |  |
| <4 | 3044 | 13.4 |
| 4-9 | 10520 | 46.5 |
| ≥10 | 9043 | 39.9 |
| **Admissions** |  |  |
| Pre-pandemic | 10173 | 45.0 |
| Pandemic: non-COVID-19 | 10982 | 48.6 |
| COVID-19 | 1452 | 6.4 |
| **Survival status** |  |  |
| Alive on discharge | 20817 | 92.1 |
| Died in hospital | 1790 | 7.9 |
